# Characterization of Tick-Borne Encephalitis Virus Isolates from *Ixodes persulcatus* Ticks Collected During 2020 in Selenge, Mongolia

**DOI:** 10.3390/pathogens13121086

**Published:** 2024-12-10

**Authors:** Bazartseren Boldbaatar, Nora G. Cleary, Julia E. Paoli, Dong-Wook Lee, Doniddemberel Altantogtokh, Graham A. Matulis, Noel Cote, Jodi Fiorenzano, Irina V. Etobayeva, Jung-Hoon Kwon, Carla Mavian, Andrew G. Letizia, Michael E. von Fricken

**Affiliations:** 1 School of Veterinary Medicine, Mongolian University of Life Sciences, Ulaanbaatar 17029, Mongolia; boldbaatar.b@muls.edu.mn; 2 Department of Environmental and Global Health, University of Florida, Gainesville, FL 32610, USA; ncleary@ufl.edu (N.G.C.); g.matulis@ufl.edu (G.A.M.); 3 Emerging Pathogens Institute, University of Florida, Gainesville, FL 32610, USA; paolij20@ufl.edu (J.E.P.); cmavian@ufl.edu (C.M.); 4 Department of Pathology, Immunology and Laboratory Medicine, University of Florida, Gainesville, FL 32610, USA; 5 College of Veterinary Medicine, Kyungpook National University, Daegu 41566, Republic of Korea; ip1003@naver.com (D.-W.L.); junghoon.kwon@knu.ac.kr (J.-H.K.); 6 National Center for Zoonotic Diseases, Ulaanbaatar 17029, Mongolia; altantogtokh@nczd.gov.mn; 7 Naval Medical Research Unit INDO PACIFIC (NAMRU-IP), Singapore 759657, Singapore; noel.m.cote.mil@health.mil (N.C.); jodi.m.fiorenzano@mil.mail (J.F.); irina.v.etobayeva.mil@health.mil (I.V.E.); andrew.g.letizia@health.mil (A.G.L.); 8 Armed Forces Pest Management Board, Silver Spring, MD 20910, USA; 9 Smithsonian Institution, Washington, DC 20024, USA

**Keywords:** tick-borne encephalitis virus, Siberian subtype, *Ixodes persulcatus*, Selenge, Mongolia, next-generation sequencing

## Abstract

Tick-borne encephalitis virus (TBEV) causes neurological disease in humans, with varied clinical severity influenced by the viral subtype. TBEV is endemic to Mongolia, where both Siberian and Far-Eastern subtypes are present. *Ixodes persulcatus* is considered the main vector of TBEV in Mongolia; although, the virus has also been detected in *Dermacentor* species. To further characterize the disease ecology of TBEV within the endemic Selenge province of Mongolia, 1300 *Ixodes persulcatus* ticks were collected in May 2020 from regions outside Ulaanbaatar. Pooled tick samples (n = 20–50) were homogenized and the supernatant was inoculated into Vero cells. Two RT-PCR assays were conducted on the cell supernatant following an observed cytopathic effect: one for TBEV detection and the second for viral subtyping. Lysed cell cultures were processed for next-generation sequencing (NGS) using Illumina technology. TBEV was detected in 10.7% of tick pools (3/28), and isolates were identified as the Siberian subtype. Phylogenetic analysis showed PQ479142 clustering within the Siberian subtype and sharing high similarity with published isolates collected in Selenge in 2012 from *Ixodes persulcatus*. Subtype analysis of circulating TBEV isolates and sequencing analytics to track viral evolution in ticks are vital to continued understanding of the risk to local populations.

## 1. Introduction

Tick-borne encephalitis (TBE) is a disease characterized by severe neurological symptoms including encephalitis and meningoencephalitis, with a case fatality rate of up to 30% [1]. This disease is caused by the tick-borne encephalitis virus (TBEV), of which five subtypes are recognized: Western, Baikalian, Himalayan, Siberian, and Far-Eastern [2,3]. While the Far-Eastern subtype is associated with serious clinical manifestations, the Siberian subtype has a lower fatality rate and is associated with chronic disease [1]. The primary vectors of the virus are members of the *Ixodes* tick genus, where these ticks also act as reservoir hosts [4].

TBEV is a member of the *Flaviviridae* family within the *Orthoflavivirus* genus, which includes other arthropod-borne viruses pathogenic to humans such as dengue and Japanese encephalitis virus [5]. TBEV is an enveloped positive-sense single-stranded RNA virus approximately 11,000 nucleotides in length [6]. The genome includes two non-coding regions at the 5′ and 3′ ends flanking a long open reading frame (ORF) which encodes for a single polyprotein [6]. The polyprotein is cleaved into three structural proteins (capsid, membrane, and envelope) and seven non-structural proteins (NS1, NS2A, NS2B, NS3, NS4A, NS4B, and NS5) [3]. Among the three structural proteins, the envelope (E) protein is most commonly used for TBEV subtyping [7]. The non-structural proteins such as NS3 and NS5 have also been used for viral subtyping, although less frequently [7].

Within Mongolia, cases of TBEV have been reported since the 1980s, with an average of 20 cases per year, although the disease may be under-reported due to a lack of awareness and diagnostic capabilities [2,8]. Serological evidence of past TBEV exposure within the Mongolian population has been reported in multiple provinces, including Arkhangai, Bayankhongor, Bulgan, Dornod, Khuvsgul, Khentii, Selenge, Sukhbaatar, Umnugobi, Ulaanbaatar, and Uvurkhangai [9,10]. Although typically asymptomatic, domestic animals have also shown evidence of previous exposure to TBEV in the Khuvsgul, Selenge, and Tuv provinces [11]. Entomological studies have demonstrated that *Ixodes persulcatus* is the primary vector for TBEV within Mongolia, with positive tick samples detected in the Bulgan and Selenge provinces [12,13,14]. Reports of TBEV in *Dermacentor nuttalli* and *Dermacentor silvarum*, suggest that the disease ecology of TBEV within Mongolia may be more complex given the wide geographic range of *Dermacentor* spp. across Mongolia, compared to the clearly defined ecological niche of *I. persulcatus*; however, vectorial competence remains unknown [9]. Tick pool positivity percentages are typically low, with often less than 8% of tick samples testing positive, with variable detection proportions based on pool size and sample region [9,12,13]. The detection of seropositive individuals within aimags (provinces) where the main *I. persulcatus* vector is not known to be present demonstrates a need for further biosurveillance within Mongolia to determine if exposure is occurring due to seasonal population movement, expansion of *I. persulcatus* habitat, or evidence of other tick-vectors responsible for disease transmission [11,15].

Sequencing analyses of TBEV isolates from Mongolia have demonstrated the presence of the Siberian and Far-Eastern subtypes, with high levels of genetic homogeneity to Russian isolates [1,2,13,16]. To further examine TBEV genetic characteristics, isolates from tick samples collected in Selenge were sequenced and analyzed.

## 2. Materials and Methods

### 2.1. Tick Collection

From 21 to 25 May 2020, ticks (n = 1300) were collected by dragging and flagging in the Eruu, Khuder, and Mandal soums (counties) of the Selenge province. All ticks were morphologically identified as *I. persulcatus* using taxonomic keys [17]. Ticks were combined into pools ranging from 20 to 50 ticks per pool based on collection location to manage costs and increase the likelihood of detection when targeting pathogens with low prevalence in vectors. The pools were placed in falcon tubes for storage at −80 °C until further processing. Pools were washed once with 70% ethanol to reduce bacterial contamination before processing.

### 2.2. TBEV PCR Detection

Vero cells were cultured at 6–7 × 10^5^ cells/mL into a 25 cm^3^ flask containing 5 mL growth medium and were allowed to grow until reaching 90% confluency. Using a mortar and pestle, tick samples were ground, and the supernatant was inoculated into a flask of confluent Vero cells cultured in maintenance medium consisting of MEM Eagle (Sigma Aldrich, St. Louis, MO, USA), 2% fetal bovine serum, penicillin, streptomycin, and Fungizone. Cells were observed daily using a light microscope for evidence of a cytopathic effect (CPE). Following an observed CPE 4–5 days post-inoculation, infected cell culture supernatant was extracted using a RNeasy kit (Qiagen, Hilden, Germany) and two different RT-PCR assays were conducted to detect the presence of TBEV. Each PCR assay product was run on a gel to confirm amplification. The first RT-PCR assay to detect TBEV used the following primers: TBEV-E-Forward 5′-GGTYATGGARGTYRCRTTCTCTGG-3′ and TBEV-E-Reverse 5′-TCCCAGGCGTGYTCTCCKATCACTGT-3′ [1]. The PCR conditions were 95 °C for 5 min, then 40 cycles of 95 °C for 30 s, 55 °C for 30 s, and 72 °C for 2 min, then held at 4 °C. The second RT-PCR assay used primers to subtype the TBEV. The Siberian E gene primers used were Forward 5′-GGTTGCCGTTGTGTGGTTGAC-3′ and Reverse 5′-TTCCGGATAGTATGCGTAGTTG-3′ with the following PCR conditions: 94 °C for 1 min, then 39 cycles of 94 °C for 1 min, 55 °C for 1 min, and 72 °C for 2 min, then held at 4 °C [11]. The Far-Eastern E gene primers used were Forward 5′-CAGAGCGGCACAGTGTGCAAGAGA-3′ and Reverse 5′-GGCCGTCGGTAGGTGTTCTG-3′. The PCR conditions for the Far-Eastern primers were 94 °C for 1 min, followed by 39 cycles of 94 °C for 30 s, 55 °C for 30 s, and 72 °C for 2 min, then 1 cycle of 4 °C for 10 min.

### 2.3. NGS Sequencing

Lysed cell cultures were filtered through 0.20 μm syringe filters (Corning Incorporated, Corning, NY, USA), and the viral genome was extracted from 100 μL of the fluid using a Qiagen RNeasy Mini Kit (Qiagen, Hilden, Germany) according to the manufacturer’s instructions. To convert extracted RNA to cDNA, the Superscript IV First-Strand Synthesis System (Invitrogen, Thermo Fisher Scientific, Waltham, MA, USA) and Large (Klenow) Fragment DNA Polymerase I (New England Biolabs, Ipswich, MA, USA) were used. A QIAquick PCR Purification Kit (QIAGEN, Hilden, Germany) was used to purify the cDNA product. Whole-genome shotgun sequencing was performed using the Illumina Next-Generation Sequencing (NGS) platform to generate TBEV genomes. Libraries were prepared using the Illumina DNA prep kit and whole-genome sequencing was accomplished using Illumina iSeq100 (Illumina, San Diego, CA, USA).

### 2.4. NGS Data Analysis

Illumina-generated FASTQ files were cleaned with Trimmomatic v0.39 to remove adapter sequences and filter out low-quality sequences (phred < 30) [18]. De novo metagenomic assembly was performed using MEGAHIT v1.1.4 [19]. The resulting five contigs were found to share high nucleotide similarity with a 2012 TBEV isolate from Mongolia (LC017692.1) according to BLASTn and BLASTx comparison [20]. Therefore, trimmed reads were aligned to LC017692.1 using Bowtie2 v2.3.5.1, and a consensus sequence was produced by incorporating only positions with a minimum read depth of five and where at least 75% of the reads agreed on the base [21]. Contigs generated by MEGAHIT were then used to refine the consensus sequence by filling in gaps where appropriate. The final consensus sequence covered 47% of the LC017692.1 full-length genome.

### 2.5. TBEV Genomic Dataset

All available complete genome sequences were downloaded for TBEV (n = 213) from NCBI Virus (https://www.ncbi.nlm.nih.gov/labs/virus), accessed on 1 October 2024, with 15 incomplete genome sequences excluded (Supplementary Table S1). An alignment was constructed using full-length NCBI sequences and the consensus genome by aligning the dataset to the TBEV reference genome available on GenBank (NC_001672.1) using MAFFT v 7.526 with default parameters [22]. The alignment was manually polished using AliView v1.28 [22,23].

### 2.6. Recombination Analyses

Recombination was assessed using RDP5 with default settings for linear sequences [24]. Statistical evidence of recombination was provided by a *p*-value of <0.05. Recombination events were identified when at least 6/7 of the following detection methods were in agreement: RDP, GENECONV, Chimaera, MaxChi, BootScan, SiScan, and 3Seq. A recombination-free alignment was obtained from RDP5 after the identification of breakpoints.

### 2.7. Pairwise Genetic Distance

Pairwise genetic distance was calculated using Molecular Evolutionary Genetics Analysis software version 11 (MEGA 11) with three different models: number of nucleotide differences, p-distance, and maximum-composite likelihood [25]. For all models, the parameters used were the Gamma distribution (shape parameter = 4.0), 100 bootstrap replicates for variance estimation, and gaps treated as complete deletions. The 1st, 2nd, 3rd, and non-coding sites were included.

### 2.8. Phylogenetic Reconstruction

A maximum likelihood (ML) phylogenetic tree was inferred with Orthoflavivirus Omsk hemorrhagic fever virus (OHFV) included as an outgroup (AY193805.1) using IQ-TREE v2.2.2.7 with the best-fit evolutionary model detected by the Bayesian information criterion (GTR+F+I+R3) and incorporating 2000 ultrafast bootstrap replicates for support [26]. Visualization of trees was performed in R using the ggtree v3.12.0 package [27].

### 2.9. Statistical Calculations

Maximum likelihood estimate (MLE) calculations and minimum infection rates (MIR) along with 95% confidence intervals for TBEV within pooled tick samples were calculated using the Centers for Disease Control and Prevention (CDC) Mosquito Surveillance Software tool at https://www.cdc.gov/mosquitoes/php/toolkit/mosquito-surveillance-software.html (accessed on 17 October 2024). MLE and MIR are calculated at a scale of 1000.

### 2.10. Mapping

Maps were generated in QGIS 3.28.1 using GPS coordinate data collected for each collection event [28]. Shapefiles for Mongolia were obtained from https://data.humdata.org/dataset/cod-ab-mng (accessed on 29 July 2024).

## 3. Results

**Figure 1:**
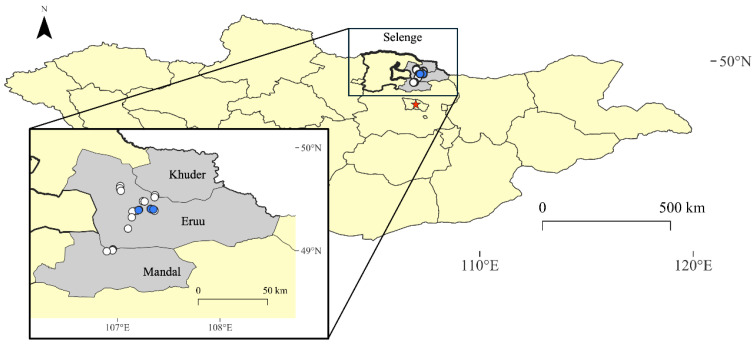
Based on PCR results, white circles represent TBEV-negative tick pools and blue circles represent TBEV-positive tick pools. The red star represents the location of the capital, Ulaanbaatar.

The Siberian subtype TBEV was detected in 3 of 28 tick pools (10.7%) by PCR. The MLE was 2.40 (95% CI 0.64, 6.56) and the MIR was 2.31 (95% CI 0.00, 4.92). Positive tick pools were exclusively from Eruu, Selenge. The geographic locations of collection events and positive samples are shown in Figure 1.

Construction of a consensus sequence using mapping Illumina reads to a reference TBEV genome (LC017692.1) and refining gaps with contigs resulted in a consensus sequence covering 47% of the reference genome length. The consensus sequence includes partial coverage of the 5′ NCR region, all structural proteins, and all non-structural proteins (Table 1). The only region of the genome not included by the consensus was the 3′ NCR.

**Figure 2:**
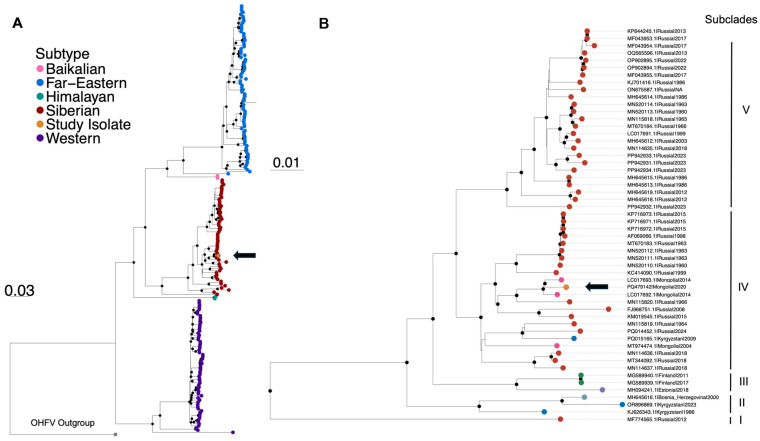
Maximum likelihood (ML) phylogenies of full-genome TBEV isolates and consensus sequence. (**A**) ML tree inferred from 198 full-genome TBEV isolates plus our consensus sequence, with OHFV as the outgroup. Tip colors indicate subtype. PQ479142 (indicated by the arrow) clusters within the Siberian subtype clade. The best-fit evolutionary model was detected by the Bayesian information criterion (GTR+F+I+R3). Black circles at nodes indicate strong statistical support at branches determined by ultrafast bootstrap support values >90. (**B**) Phylogeny of Siberian clade. Tip color indicates geographic origin. Five Siberian subclades as defined by monophyletic clades with branch support > 90 are indicated. All isolates collected in Mongolia belong to Subclade IV. PQ479142 (indicated by the arrow) clusters with two isolates collected in Selenge, Mongolia in 2012 from *I. persulcatus* (LC017692.1 and LC017693.1).

Recombination analysis detected 13 events impacting TBEV subtypes; however, there was no evidence that our isolate PQ479142 was recombinant (Supplementary Table S2). Likelihood mapping revealed a low percentage of unresolved quartets (1%), indicating sufficiently robust phylogenetic signal in the alignment to proceed with further analysis (Figure S1). Maximum likelihood trees inferred from the recombinant-free alignment and rooted with the outgroup Omsk hemorrhagic fever virus show distinct clades for the five subtypes with strong branch support for each clade: Far Eastern, Baikalian, Siberian, Himalayan, and Western (Figure 2A). Our consensus sequence clusters within the Siberian subtype that includes isolates from Mongolia, Russia, Estonia, Finland, Bosnia and Herzegovina, and Kyrgyzstan (Figure 2B). The Siberian subtype comprises highly divergent isolates from different countries, showing no geographic compartmentalization and forming five subclades, as defined by monophyletic clades with branch support of >90 (Figure 2B).

Starting from the bottom of the tree, subclade I consists of one isolate collected in Russia in 2012, which is a known new lineage of the Siberian subtype and forms a remote outgroup (GenBank ID MF774565.1) [29]. Subclade II consists of three isolates, one from Bosnia-Herzegovina (collection year 2000) and two from Kyrgyzstan (collection years 1986, 2023), which have previously been described as clustering together [30]. Subclade III is formed by two isolates from Finland (collection years 2011, 2017) and one from Estonia (collection year 2018). Subclade IV is formed of 24 isolates ranging in collection years from 1960 to 2024 from Russia, Kyrgyzstan, and all Mongolian samples, including our isolate. Finally, subclade V consists entirely of isolates from Russia collected between 1960 and 2023. Maximum likelihood trees for individual contigs coding for different regions of the genome also showed PQ479142 clustering in the same pattern as LC017692.1 and LC017693.1 in subclade IV (Supplementary Figure S2a–e).

Within Siberian subclade IV, PQ479142 clusters with high branch support together with two Mongolian isolates collected in 2012, LC017692.1 and LC017693, belonging to the strain MGL-Selenge-13. The 2012 Mongolian isolates were collected in the same northern aimag, Selenge, and from the same tick species, *I. persulcatus*, as PQ479142. The 2020 Mongolian isolate shares 98.9% nucleotide identity with LC017692.1 and 98.8% nucleotide identity with LC017693.1 (Supplementary Table S3).

## 4. Discussion

Within this study, TBEV was detected in 10.7% of *I. persulcatus* tick pools collected in the Selenge aimag. The observed tick pool positivity rate is higher than in previous studies that reported an individual tick positivity percentage of 1.3% from pools of 20–30 in *I. persulcatus* collected from Selenge [1]. This discrepancy is likely due to the larger pool sizes in our study which could lead to an overestimate of pool positivity. The MIR of 2.31 in our study is a more conservative representation of positive TBEV detections indicating that approximately 2.3 ticks per 1000 are infected with TBEV. Based on the MLE, the infection rate of TBEV among 1000 ticks is 0.24%, providing further insight into our pooled analysis. Despite these low calculated infection rates, the clinical relevance of these findings cannot be ignored, given that the Selenge population has the highest TBEV seroprevalence within Mongolia, suggesting frequent exposure [31]. It is likely that this has led to the Selenge aimag reporting a 2.7% case fatality rate from 2008 to 2017 [32]. The TBEV isolates in this study show high sequence similarity to the Siberian subtype which has been previously documented in *I. persulcatus* ticks from Selenge province [1,16,32]. This ongoing predominance of the Siberian subtype within Selenge is in accordance with the aimag’s reported low TBEV mortality rate, given that higher mortality rates are associated with the Far-Eastern subtype.

While recombination is known to occur between TBEV viruses in close geographic vicinity, in-depth sequence analyses of PQ479142 demonstrated that it is not recombinant [32]. Within the Siberian clade, subclade IV, there was strong branch support for PQ479142 clustering with two 2012 isolates collected in the same province and from the same species of ticks: LC017692.1 and LC017693.1. Despite being collected eight years apart, the three Selenge isolates have high similarity at the nucleotide level (47% across the genome). However, the long branch connecting PQ479142 to the previous ones, and therefore the distant ancestor node, is somewhat suggesting that TBEV has been slowly evolving in ticks and/or cryptic circulation in reservoir(s). TBEV strains in the same geographic region are known to be highly genetically similar [33]. In a previous study, TBEV was found to have the lowest rate of substitution among all tick-borne flaviviruses which include Powassan virus, Omsk hemorrhagic fever virus, and Kyasanur Forest disease virus [34]. Factors which may contribute to a slow rate of evolution within the *I. persulcatus* population include the persistence of TBEV within ticks throughout the tick’s life cycle and the low prevalence of TBEV in tick populations [35]. Among *Ixodes* with persistent TBEV infection, experimental studies have shown that the genetic makeup of viral populations remains stable over time [36]. In addition to a relatively slow substitution rate, TBEV viruses are also known to spread slowly across geographic regions, so it is not surprising to find genetically similar isolates collected in the same province years apart [32]. Another factor likely contributing to the stability of TBEV over time is the low infection rate among *I. persulcatus* in Selenge, ranging from 1.3–1.6% [1,2]. Given the high genetic stability of TBEV from 2014 to 2020, additional analyses utilizing genome isolate sequences with larger reference length coverage would provide further insight into regional viral evolution over time.

Subtyping TBEV isolates from ticks can provide insights into disease risk among humans residing in that area. While both Siberian and Far-Eastern subtypes have been found in Mongolia, current vaccines have been individually developed based on the Western and Far-Eastern subtypes. While no vaccine has been specifically developed for the Siberian subtype, the existing vaccines have been shown to provide some cross-reactive immunity to other TBEV subtypes [37]. The continued detection of the Siberian subtype in ticks from Selenge poses a risk to the local population, possibly including those who have been vaccinated, although more studies are needed to better understand the cross-immunity afforded to Mongolian isolates of the Siberian subtype by these vaccines [37]. Further genomic analysis of wild-type TBEV, especially in Selenge, can inform future vaccine development and prevention strategies.

Researchers should discuss the results and how they can be interpreted from the perspective of previous studies and of the working hypotheses. The findings and their implications should be discussed in the broadest context possible. Future research directions may also be highlighted.

## 5. Limitations

The morphological identification of tick species may have led to misidentifications, although current tick ecological data suggests that *I. persulcatus* is the only *Ixodes* species present within Selenge [18]. In the construction of the consensus sequence, TBEV contigs were assembled to obtain one sequence from pools, because we assumed the contigs were obtained from an individual tick, given that around 1.3–1.6% of ticks are infected with TBEV [1,2]. The decision to pool ticks within this study precludes our results from being used to determine the true prevalence of TBEV within the *I. persulcatus* tick populations of Selenge. The collection period was limited to five days; therefore, this analysis may not be representative of TBEV disease ecology during other times throughout the year. Pooling may reduce the sensitivity of viral detection assays and limit the usefulness of prevalence data aside from identifying viral presence in specific locations. Tick density was not calculated due to the lack of feasibility and reliability.

## 6. Conclusions

In Selenge, ticks remain a threat for TBEV transmission, especially the Siberian subtype, despite no significant genetic evolution being demonstrated between sampling occurrences in 2012 and 2020. Given the established presence in Selenge and previous documentation of TBE infection in humans and ticks in this province, ongoing monitoring and molecular characterization of TBEV in Mongolia is necessary to ensure that appropriate public health interventions continue to be applied to protect local populations.

## Figures and Tables

**Table 1:** Comparison of consensus genome to reference genome. The nucleotide lengths (number of base pairs) of the 5′ NCR, structural genes, non-structural genes, and 3′ NCR were compared between the reference genome used for mapping (LC017692.1) and our consensus sequence. The percentage of each genome region covered by the consensus was calculated by dividing the length of the consensus sequence by the length of the reference sequence.

Genome Region	Length of PQ479142 (# bp)	Length of LC017692.1 (# bp)	% of Region Included by Consensus
5′ NCR	115	132	87
C	331	336	99
prM	203	504	40
E	343	1488	23
NS1	261	1056	25
NS2a	209	690	30
NS2b	28	393	7
NS3	1611	1863	86
NS4a	337	378	89
2k	69	69	100
NS4b	406	756	54
RDRP	1297	2709	48
3′ NCR	0	732	0
Total	5210	11,106	47

# bp: number of base pairs; 5′ NCR: 5′ non-coding region; C: capsid protein; prM: pre-membrane protein; E: envelope protein; NS1: non-structural protein 1; NS2a: non-structural protein 2a; NS2b: non-structural protein 2b; NS3: non-structural protein 3; NS4a: non-structural protein 4a; 2k: 2k protein; NS4b: non-structural protein 4b; RDRP: RNA-dependent RNA polymerase; 3′ NCR: 3′ non-coding region.

## Data Availability

Dataset available on request from the authors.

## Supplementary Materials

The following supporting information can be downloaded at: https://www.mdpi.com/article/10.3390/pathogens13121086/s1, Figure S1: Likelihood map to assess phylogenetic signal; Figure S2: Siberian clade maximum likelihood trees for contigs; Table S1: List of accessions in TBEV dataset; Table S2: Recombinant events identified by RDP5 for the TBEV dataset; Table S3: Pairwise genetic distance comparison of our 2020 Mongolian isolate and two 2012 isolates from Mongolia.

## Disclaimer

The views expressed are those of the author(s) and do not necessarily reflect the official policy or position of the Department of the Navy, Department of Defense, nor the U.S. Government. Copyright Statement: LCDR Noel Cote (MSC, UNS), LCDR Jodi Fiorenzano (MSC, USN) & CAPT Andrew G. Letizia (MC, USN) are military service members and employees of the U.S. Government. This work was prepared as part of their official duties at U.S. Naval Medical Research Unit INDO PACIFIC. Title 17 U.S.C. §105 provides that ‘Copyright protection under this title is not available for any work of the United States Government.’ Title 17 U.S.C. §101 defines a U.S. Government work as a work prepared by a military service member or employee of the U.S. Government as part of that person’s official duties.
