# Supplementary material for: Characterization of Tick-Borne Encephalitis Virus Isolates from Ixodes persulcatus Ticks Collected During 2020 in Selenge, Mongolia
Source: Pathogens. 2024 Dec 10;13(12):1086. doi: 10.3390/pathogens13121086 (PMC11677234; doi:10.3390/pathogens13121086)
Supplement: Supplementary file 1 [file pathogens-13-01086-s001.zip › pathogens-3350499-supplementary.pdf]

SUPPLEMENTARY TABLES

Table S1. List of Accessions in TBEV Dataset

GenBank Accession Number	Collection Year	TBEV Subtype
KJ633033.1	1984	Baikalian
EF469662.1	1984	Baikalian
PP947711.1	1993	Far-Eastern
PP708890.1	1999	Far-Eastern
PP947713.1	1999	Far-Eastern
PP937588.1	1985	Far-Eastern
EU816455.2	1986	Far-Eastern
FJ997899.1	1990	Far-Eastern
EU816452.1	1991	Far-Eastern
PP708883.1	1982	Far-Eastern
EU816450.1	1991	Far-Eastern
AY169390.3	1991	Far-Eastern
PP708888.1	1984	Far-Eastern
EU816451.1	1991	Far-Eastern
KU761567.1	1958	Far-Eastern
KU761574.1	1958	Far-Eastern
GQ228395.1	1997	Far-Eastern
EU816453.1	2000	Far-Eastern
AB753012.1	2008	Far-Eastern
PP947712.1	1987	Far-Eastern
PP937586.1	1991	Far-Eastern
PP937587.1	1992	Far-Eastern
PP708884.1	1983	Far-Eastern
FJ402886.1	1973	Far-Eastern
PP937585.1	1991	Far-Eastern
HQ901366.1	2009	Far-Eastern
HQ901367.1	2010	Far-Eastern
HM859895.1	1985	Far-Eastern
PP708887.1	1991	Far-Eastern
HQ201303.1	1992	Far-Eastern
EU816454.1	1994	Far-Eastern
MT671302.1	1957	Far-Eastern
PP708885.1	1983	Far-Eastern
PP708886.1	1982	Far-Eastern

PP708889.1	1978	Far-Eastern
FJ906622.1	1987	Far-Eastern
HM859894.1	1978	Far-Eastern
KT001070.1	2013	Far-Eastern
KP844726.1	2013	Far-Eastern
KP844727.1	2013	Far-Eastern
KT001071.1	2013	Far-Eastern
KT001072.1	2013	Far-Eastern
KP844724.1	2012	Far-Eastern
KP844725.1	2012	Far-Eastern
KT069219.1	1958	Far-Eastern
KU761571.1	1958	Far-Eastern
KU761569.1	1960	Far-Eastern
OP037818.1	NA	Far-Eastern
KU761576.1	1937	Far-Eastern
JF819648.2	1937	Far-Eastern
MT671301.1	1969	Far-Eastern
MN115817.1	1969	Far-Eastern
KF951037.1	1966	Far-Eastern
MT671300.1	1962	Far-Eastern
LC440459.1	2017	Far-Eastern
KP869172.1	1985	Far-Eastern
KU761570.1	1958	Far-Eastern
KU761572.1	1958	Far-Eastern
KU761573.1	1958	Far-Eastern
KU761575.1	1958	Far-Eastern
KJ914682.1	2008	Far-Eastern
KJ739731.1	2006	Far-Eastern
KJ739729.1	2008	Far-Eastern
KJ914683.1	2008	Far-Eastern
KJ739730.1	2008	Far-Eastern
KM019546.1	2015	Far-Eastern
LC440460.1	2018	Far-Eastern
GU121642.1	2008	Far-Eastern
KJ755186.1	2012	Far-Eastern
JX534167.1	2012	Far-Eastern
JQ650522.1	2001	Far-Eastern
JQ650523.1	1953	Far-Eastern
JF316707.1	2010	Far-Eastern

JF316708.1	2010	Far-Eastern
KU761568.1	1959	Far-Eastern
FJ402885.1	1985	Far-Eastern
ON408072.1	NA	Far-Eastern
KT001073.1	2014	Far-Eastern
ON408071.1	NA	Far-Eastern
ON408073.1	2021	Far-Eastern
EF469661.1	1979	Far-Eastern
MG599476.1	2013	Himalayan
MG599477.1	2013	Himalayan
6_S6	2020	Siberian
LC017692.1	2014	Siberian
LC017693.1	2014	Siberian
MT670183.1	1963	Siberian
AF069066.1	1998	Siberian
KP716971.1	2015	Siberian
KP716973.1	2015	Siberian
KP716972.1	2015	Siberian
MN520110.1	1960	Siberian
MN520111.1	1963	Siberian
MN520112.1	1963	Siberian
KM019545.1	2015	Siberian
MT344092.1	2018	Siberian
MN114636.1	2018	Siberian
MN114637.1	2018	Siberian
KC414090.1	1999	Siberian
MT974474.1	2004	Siberian
PQ015165.1	2009	Siberian
PQ014452.1	2024	Siberian
MN115819.1	1964	Siberian
MN115820.1	1966	Siberian
FJ968751.1	2008	Siberian
PP942931.1	2023	Siberian
PP942933.1	2023	Siberian
PP942934.1	2023	Siberian
MT670184.1	1966	Siberian
MN520113.1	1960	Siberian
MN520114.1	1963	Siberian
MN115818.1	1965	Siberian

MN114635.1	2018	Siberian
MH645612.1	2003	Siberian
LC017691.1	1999	Siberian
MH645614.1	1986	Siberian
OQ565596.1	2013	Siberian
OP902894.1	2022	Siberian
MF043955.1	2017	Siberian
OP902895.1	2022	Siberian
MF043953.1	2017	Siberian
KP644245.1	2013	Siberian
MF043954.1	2017	Siberian
KJ701416.1	1986	Siberian
ON675587.1	NA	Siberian
MH645613.1	1986	Siberian
MH645615.1	1986	Siberian
MH645618.1	2012	Siberian
MH645619.1	2012	Siberian
PP942932.1	2023	Siberian
MG589939.1	2017	Siberian
MG589940.1	2011	Siberian
KJ626343.1	1986	Siberian
MH094241.1	2018	Siberian
OR896869.1	2023	Siberian
MH645616.1	2000	Siberian
MF774565.1	2012	Siberian
NC_001672.1	NA	Western
OL441148.1	2020	Western
MW256716.1	1952	Western
MG210945.1	2011	Western
MG210946.1	2012	Western
MG210947.1	2012	Western
MG210948.1	2016	Western
KU885457.1	1951	Western
KJ000002.1	1951	Western
MT581212.1	2019	Western
OP037819.1	NA	Western
MT228625.1	2020	Western
MT228626.1	2020	Western
MT228627.1	2020	Western

MT228628.1	2020	Western
KP716975.1	2015	Western
KP716974.1	2015	Western
KP716976.1	2015	Western
KP716977.1	2015	Western
KP716978.1	2015	Western
KF151173.1	1990	Western
OQ435379.1	1987	Western
MG589937.1	2015	Western
MG589938.1	2017	Western
MK801809.1	2017	Western
MK801813.1	2018	Western
KC835595.1	1980	Western
KC835597.1	1990	Western
KC835596.1	1990	Western
MK801803.1	2005	Western
MK560446.1	1968	Western
MK562430.1	1967	Western
KP331441.1	2008	Western
KP331442.1	2009	Western
KP331443.1	2009	Western
KP938507.1	2010	Western
FJ572210.1	2006	Western
HM535610.1	2006	Western
HM535611.1	2006	Western
KX268728.1	2011	Western
KJ922514.1	1953	Western
GQ266392.1	2005	Western
KJ922512.1	1953	Western
MK801804.1	2013	Western
MK801808.1	2013	Western
MK801805.1	2013	Western
MK801806.1	2013	Western
OR523238.1	2019	Western
KJ922516.1	1953	Western
KJ922513.1	1953	Western
KJ922515.1	1953	Western
MN735988.1	2019	Western
MN735990.1	2019	Western

MN735989.1	2019	Western
MN128700.1	2018	Western
KX966398.1	2008	Western
DQ401140.3	2003	Western
KX966399.1	2008	Western
MN735991.1	2009	Western
MN661145.1	2019	Western
LC171402.1	2015	Western
MG243699.1	2017	Western

Table S2. Recombinant events identified by RDP5 for the TBEV dataset. Recombinant sequences and their corresponding minor and major parents are listed. Statistical evidence of recombination given for six methods with a p-value < 0.05 considered significant. NS indicates lack of significant statistical evidence. Breakpoint start and end positions relative to the alignment are given.

Recombination				RDP5 Detection Method							Breakpoint Position	
Event	Recombinant	Minor Parent	Major Parent	RDP	GENECONV	Bootscan	Maxchi	Chimaera	SiSscan	3Seq	Start	End
1	KP716977.1	AF069066.1	KP716974.1	NS	1.43E-201	4.08E-191	2.20E-39	3.27E-39	2.67E-44	5.06E-246	488	2442
2	KP716972.1	OP037819.1	AF069066.1	NS	7.14E-195	1.57E-186	4.53E-39	8.27E-39	2.98E-45	7.80E-246	492	2452
3	KP716971.1	OP037819.1	AF069066.1	NS	2.60E-173	5.45E-116	3.22E-33	6.39E-33	1.44E-38	2.12E-187	968	2452
4	KP716976.1	AF069066.1	KP716974.1	NS	2.23E-178	6.36E-165	2.82E-33	5.49E-33	2.10E-35	5.97E-188	977	2442
5	KP716978.1	AF069066.1	OP037819.1	6.35E-96	7.86E-69	5.46E-102	9.34E-37	7.16E-37	5.22E-103	7.20E-255	2441	11111
6	KP716973.1	KP716978.1	AF069066.1	4.61E-125	3.49E-59	1.84E-102	7.48E-37	3.01E-36	1.56E-105	6.83E-289	2451	11082
7	PP937585.1	GQ228395.1	PP708884.1	3.13E-15	2.47E-02	3.99E-08	1.89E-05	1.57E-05	1.31E-02	6.63E-10	3330	4510
8	MN115820.1	MT670184.1	LC017693.1	9.07E-09	1.20E-08	2.84E-07	1.38E-03	1.06E-03	6.58E-04	4.51E-06	6408	6934
9	KJ626343.1	KP716972.1	MH645616.1	4.57E-04	4.82E-03	NS	2.99E-04	1.25E-04	2.84E-02	3.11E-03	7209	7672
10	JQ650522.1	JF316708.1	JX534167.1	6.93E-12	4.17E-10	NS	8.04E-05	3.56E-04	4.26E-04	3.18E-09	8067	9255
11	OP037818.1	FJ968751.1	KF951037.1	1.29E-38	1.11E-31	3.72E-16	1.05E-06	3.50E-05	1.09E-08	3.08E-03	10385	10775
12	EU816454.1	OR896869.1	HQ901366.1	8.21E-27	1.11E-24	1.81E-22	6.86E-07	5.38E-04	8.55E-09	NS	10448	10790
13	MK560446.1	GU121642.1	HM535610.1	1.15E-22	1.08E-13	2.06E-18	5.57E-07	6.56E-05	5.67E-05	6.63E-11	10549	10646

Table S3. Pairwise genetic distance comparison of our 2020 Mongolian isolate and two 2012 isolates from Mongolia. Given by three models: number of nucleotide differences, *p*-distance, and maximum composite likelihood. For all models gaps were treated as complete deletions. Codon positions 1-3 and noncoding sites were included.

	Pairwise Distance		
**Comparison Sequences**	**# of Nucleotide Differences**	**P-Distance (SE)**	**Maximum Composite Likelihood (SE)**
LC017692.1 and LC017693.1	48	0.00922 (0.00134)	0.00933 (0.00150)
6_S6 and LC017692.1	54	0.01037 (0.00132)	0.01051 (0.00160)
6_S6 and LC017693.1	56	0.01075 (0.00140)	0.01089 (0.00166)

SUPPLEMENTARY FIGURES

Figure S1

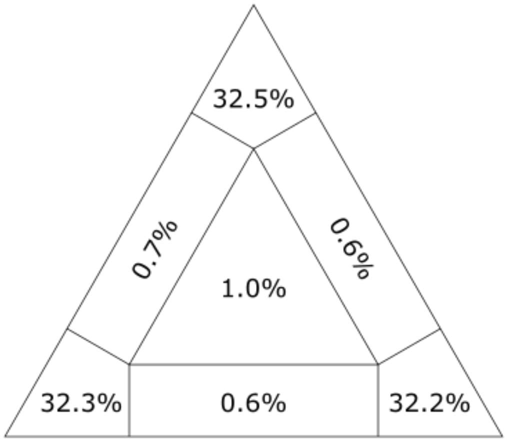

Figure S1. Likelihood map to assess phylogenetic signal. Phylogenetic signal of the recombinant-free MSA including 198 full-length TBEV genomes, our consensus genome, and the full-length genome of the OHFV outgroup. Phylogenetic signal was assessed by likelihood mapping checking for alternative topologies (tips), unresolved quartets (center), and partly resolved quartets (edges).

Figure S2

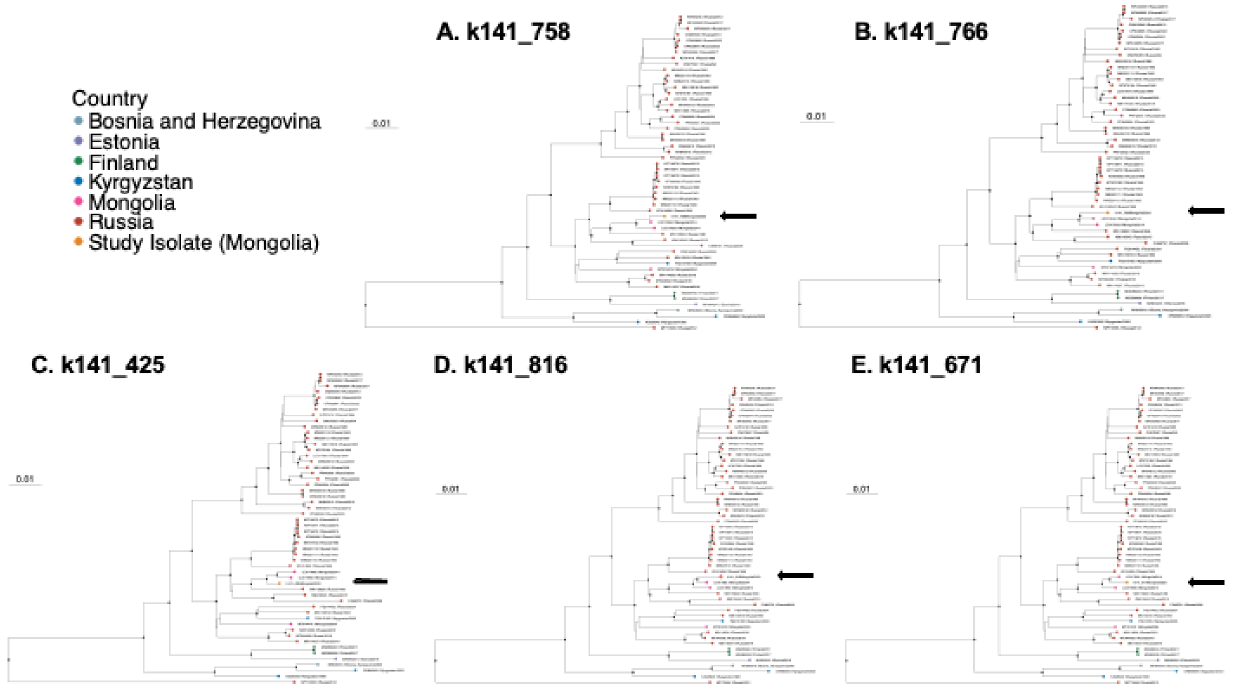

Figure S2. Siberian clade maximum likelihood trees for contigs. Maximum likelihood (ML) trees for five contigs generated by *de novo* metagenomic assembly. For each contig, ML trees were inferred from a recombinant-free MSA including 198 TBEV complete genomes and rooted with the outgroup OHFV. Tips colored according to location and black circles represent branches supported by ultrafast bootstrap (2000 replicates) > 0.90. Like our consensus sequence, each contig belongs to the Siberian subtype clade and clusters with the 2012 isolates from Mongolia LC017692.1 and LC017693.1. Contigs indicated by arrow. **(A)** Contig k141_758 (446 bp) coding for capsid protein. **(B)** Contig k141_766 (1664 bp) coding for NS3. **(C)** Contig k141_425 (713 bp) coding for NS4a and NS4b. **(D)** Contig k141_816 (305 bp) coding for NS5. **(E)** Contig k141_671 (594 bp) coding for NS5.
